# Genome-wide survey identified superior and rare haplotypes for plant height in the north-eastern soybean germplasm of China

**DOI:** 10.1007/s11032-023-01363-7

**Published:** 2023-03-20

**Authors:** Hui Yu, Javaid Akhter Bhat, Candong Li, Beifang Zhao, Tai Guo, Xianzhong Feng

**Affiliations:** 1 Key Laboratory of Soybean Molecular Design Breeding, Northeast Institute of Geography and Agroecology, Chinese Academy of Sciences, Changchun, 130102 China; 2 Zhejiang Lab, Hangzhou, 310012 China; 3 Jiamusi Branch Academy of Heilongjiang Academy of Agricultural Sciences, Jiamusi, 154007 China

**Keywords:** Soybean, Plant height, GWAS, Haplotype, Improvement

## Abstract

The proper and efficient utilization of natural genetic diversity can significantly impact crop improvements. Plant height is a quantitative trait governing the plant type as well as the yield and quality of soybean. Here, we used a combined approach including a genome-wide association study (GWAS) and haplotype and candidate gene analyses to explore the genetic basis of plant height in diverse natural soybean populations. For the GWAS analysis, we used the whole-genome resequencing data of 196 diverse soybean cultivars collected from different accumulated temperature zones of north-eastern China to detect the significant single-nucleotide polymorphisms (SNPs) associated with plant height across three environments (E1, E2, and E3). A total of 33 SNPs distributed on four chromosomes, viz., Chr.02, Chr.04, Chr.06, and Chr.19, were identified to be significantly associated with plant height across the three environments. Among them, 23 were consistently detected in two or more environments and the remaining 10 were identified in only one environment. Interestingly, all the significant SNPs detected on the respective chromosomes fell within the physical interval of linkage disequilibrium (LD) decay (± 38.9 kb). Hence, these genomic regions were considered to be four quantitative trait loci (QTLs), viz., *qPH2*, *qPH4*, *qPH6*, and *qPH19*, regulating plant height. Moreover, the genomic region flanking all significant SNPs on four chromosomes exhibited strong LD. These significant SNPs thus formed four haplotype blocks, viz., Hap-2, Hap-4, Hap-6, and Hap-19. The number of haplotype alleles underlying each block varied from four to six, and these alleles regulate the different phenotypes of plant height ranging from dwarf to extra-tall heights. Nine candidate genes were identified within the four haplotype blocks, and these genes were considered putative candidates regulating soybean plant height. Hence, these stable QTLs, superior haplotypes, and candidate genes (after proper validation) can be deployed for the development of soybean cultivars with desirable plant heights.

**Supplementary Information:**

The online version contains supplementary material available at 10.1007/s11032-023-01363-7.

## Introduction

Soybean (*Glycine max* L.) is a wealthy source of edible oil and protein and is cultivated globally (Hyten et al. 2004). Current statistics show that China is mostly dependent on imported soybean and soybean products (Liu et al. 2018), and the yield improvement of soybean has been almost stagnant within the country over the past 50 years (Bhat et al. 2022). In this regard, plant architecture is one of the important determinants of soybean yield, and plant height is an important quantitative trait regulating the soybean plant type. Therefore, the development of soybean cultivars with desirable plant heights is among the major objectives of soybean breeding programs. Studies have documented the significant correlation between plant height and yield in soybean (Lu et al. 2016). Hence, for the development of soybean cultivars with desirable plant heights, it is a prerequisite to first elucidate the detailed genetic makeup affecting the plant height. Understanding the genes regulating plant height can allow the direct introduction of these genes into the marker-assisted breeding program pipeline to develop soybean cultivars with desirable plant heights (Bhat et al. 2021).

In the past few decades, efforts have been made to unravel the genetic basis of plant height in soybean, and > 200 genetic loci associated with plant height have been documented in SoyBase (https://www.soybase.org/). These documented quantitative trait loci (QTLs) were mostly identified using the low-resolution approach of traditional linkage mapping and low-density marker systems. Hence, the disadvantages associated with such low-resolution information have restricted their deployment in the breeding of desirable soybean plant types (Cao et al. 2019). To this end, the genome-wide association mapping (GWAS) method, which possesses the attributes of a high resolution and high accuracy, has been seen as a more practical approach for marker-assisted breeding applications (Schmutz et al. 2010; Bhat et al. 2021). The practical application of the GWAS method in crop/soybean gene mapping applications has been verified by recent advances in genome sequencing and high-throughput genotyping, which have allowed its routine use in crop plants (Song et al. 2016; Cao et al. 2019). The performance of GWAS in gene mapping has been demonstrated by multiple studies focusing on various crops such as maize (Yang and Zhu 2005), soybean (Chang et al. 2018; Bhat et al. 2022), chickpea (Thudi et al. 2021), *Arabidopsis thaliana* (Ren et al. 2019), rice (Li et al. 2014), and wheat (Wang et al. 1999).

It is well proven that GWAS has great potential in elucidating the genetic basis of complex crop plant traits. In soybean, most single-nucleotide polymorphism (SNP) markers have been used in GWAS analysis studies. However, the biallelic nature of SNP markers decreases their efficiency in GWAS analyses, and the use of these markers often leads to the loss of superior and rare alleles regulating the desirable phenotype of a particular crop trait (Bhat et al. 2021). Many studies have used empirical and simulation methods to check the efficiency of haplotype markers in GWAS, revealing the better performance of haplotype markers relative to SNP markers (Lu et al. 2012; N’Diaye et al. 2017; Luján Basile et al. 2019). This superiority of haplotype markers relative to SNPs in GWAS and genomic selection (GS) analyses is the result of multiple features, such as their multiallelic nature, the considerably higher number of haplotype variants, their higher polymorphism contents, the better control of false-positives and false-negatives, the identification of rare alleles, and their abilities to capture epistatic variations (Bhat et al. 2021). Recently, the use of haplotype markers has been observed to enhance the allelic effect and phenotypic variation explained (PVE) by 34% and 50%, respectively, compared to the use of SNP markers (Hamblin and Jannink 2011). Similarly, in wheat crops, the average polymorphism information content (PIC) is enhanced by 0.50 when using haplotype markers compared to 0.27 when using SNPs (N’Diaye et al. 2017). Moreover, the successful use of *haplopheno* analyses in studies identifying superior haplotypes for economically important agronomic traits in crop plants has been reported. For instance, 3000 rice accession panels were used in a GWAS analysis study to identify the superior haplotypes underlying 21 genes controlling quality and yield-related traits in rice (Abbai et al. 2019). Moreover, desirable haplotypes for abiotic stress tolerance have been identified in different crop species, viz., soybean, rice, and pigeonpea (Guan et al. 2014; Kuroha et al. 2018; Sinha et al. 2020a, b).

To elucidate the genetic makeup of plant height in soybean, we used diverse soybean germplasms collected from the different accumulated temperature zones of north-eastern China to conduct GWAS, haplotype, and candidate gene analyses. The current study identified the major stable QTLs, haplotypes, and candidate genes associated with soybean plant height. The QTLs and haplotypes identified herein can be deployed to breed soybean crops with desirable plant heights, and the identified candidate genes can be used to improve soybean crops following their proper functional validation.

## Materials and methods

### Plant materials and field experiments

A panel of 196 diverse soybean cultivars collected from five accumulated temperature zones (in each zone, the temperature remains constant) of the north-eastern parts of China was used as the plant material data in the current study. The soybean germplasm was evaluated at the Jiamusi Experimental Research Farm located in a prefecture-level city in eastern Heilongjiang Province of China in three consecutive years, viz., 2017, 2018, and 2019, representing three environments, E1, E2, and E3, respectively. Jiamusi (45.7421° N, 126.6629° E) has a monsoon-influenced humid continental climate (Köppen: Dwa) and possesses dry, bitter, and long winters, as well as very warm summers. This location has an average relative humidity, average rainfall, and average temperature of 62.8%, 725.3 mm, and 15.4 °C, respectively. The randomized complete block design (RCBD) was followed in triplicate when planting this soybean germplasm. The plot possessed three rows of each genotype with a row length and spacing of 200 and 50 cm, respectively. For the cultivation of the soybean germplasm, the recommended agronomic practices were followed in each year.

### Phenotypic data collection and analysis

For the collection of plant height phenotypic data, five plants were randomly selected from the middle of each plot at the maturity stage. The length from the cotyledonary node to the peak of the main stem represents the plant height. The average plant height taken from the five plants of each genotype across the triplicate plots in each year/environment was used for the analysis.

Parameters of descriptive statistics, viz., the range, mean, standard deviation (SD), skewness, kurtosis, and coefficient of variation (CV%), of the plant height were calculated using R software, and the specific function with proper summary statistics was used. The *aov* function of R software was used to perform an analysis of variance (ANOVA). Heritability (*h*^2^) was estimated in a broad sense by using the equation below:

$${h}^{2}={{\sigma }^{2}}_{\mathrm{g}}/\left({{\sigma }^{2}}_{\mathrm{g}}+{{\sigma }^{2}}_{\mathrm{ge}}/n+{{\sigma }^{2}}_{\mathrm{e}}/nr\right)$$

where *σ*^2^_*g*_ represents the genotypic variance, *σ*^2^_*ge*_ represents the genotype-by-environment interaction variance, *σ*^2^_*e*_ corresponds to the error variance, *n* represents the number of environments, and *r* represents the number of replications (Nyquist and Baker 1991).

### Resequencing analysis and genotyping

The genomic DNA corresponding to all soybean accessions was extracted from fresh and healthy soybean leaves after 3 weeks of growth. DNA extraction was performed using the cetyltrimethylammonium bromide (CTAB) method (Murray and Thompson 1980). A library containing each accession was constructed with an insert size of approximately 350 base pairs by following the manufacturer’s instructions (Illumina Inc., San Diego, CA, USA). The resequencing of the 196 soybean accessions was carried out by the Illumina HiSeq platform. We performed a stringent quality control analysis to select high-quality SNPs by using the parameters of minor allele frequency (MAF) of 0.05 and missing genotype frequency of 0.10 in VCFtools software v0.1.13 (Danecek et al. 2011); finally, we identified a total of 4,665,814 high-quality SNPs that were further used in the GWAS analysis.

### Linkage disequilibrium (LD) and genome-wide association study (GWAS)

To estimate the genome-wide LD, PopLDdecay software (Zhang et al. 2019) was used to calculate the *r*^2^ (squared allele-frequency correlations) values among the SNPs with known genomic positions. PopLDdecay software (Zhang et al. 2019) was also used to estimate the *r*^2^ (expected values) under drift equilibrium, and the same results were plotted against the physical distance (kb) using PopLDdecay software. The LD decay curve was fitted on the scatterplot by smoothing the spline regression line at the genome level (Remington et al. 2001).

The GWAS analysis was conducted with Trait Analysis by Association, Evolution, and Linkage v5.2.73 (TASSEL v5.2.73) software (Bradbury et al. 2007) by using a compressed mixed linear model (CMLM), and the default parameters, viz., a filterAlignMinCount value of 1 and filterAlignMinFreq value of 0.05, were used for the GWAS screening in TASSEL v5.2.73. This model possesses a higher statistical power than other models for detecting the association of markers with traits of interest (Zhang et al. 2010). In the CMLM, individuals are grouped into clusters, and in the mathematical model, the genetic values of the clusters are derived by fitting random effects (Zhang et al. 2010). In addition, this model decreases the computation time and provides an optimum performance at the statistical level (Bradbury et al. 2007).

### Haplotype analysis

Haploview 4.2 (Barrett et al. 2005) was used to estimate the LD levels among the SNP pairs. All the significant SNP markers identified on Chr.02, Chr.04, Chr.06, and Chr.19 fell within LD ± 38.9 kb. Hence, all these SNPs were considered in the haplotype block analysis, and the “confidence intervals” algorithm was used to define the haplotype block (Gabriel et al. 2002). Based on the specific haplotype allele carried by each genotype, the genotypes of the soybean panel were grouped into separate groups. A one-way analysis of variance (ANOVA) model was used to fit the groups for estimating the haplotype effect on plant height as follows:

$$\mathrm{model}\leftarrow \mathrm{aov}\left(\mathrm{phenotype}\sim \mathrm{group},\mathrm{ data}=\mathrm{data}\right)$$

where the plant height in the combined environment represents the phenotype. Tukey’s HSD test was used to perform pairwise comparisons of the means, and the results were visualized in the R environment.

### Candidate gene identification

All the model genes and their functional annotations present within the haplotype block were retrieved online from SoyBase (https://www.soybase.org/) using the Williams 82 (*Wm82.a2.v1*) gene model. In addition, freely available RNA-seq data representing the different soybean tissues at different growth stages in SoyBase (https://www.soybase.org/) were also retrieved for the model genes. This RNA-seq dataset was deposited in the SoyBase by Severin et al. (2010).

## Results

### Phenotypic evaluation of plant height in the GWAS panel

The descriptive statistics analysis results of plant height in the soybean panel, viz., the maximum value, minimum value, SD, CV, kurtosis, skewness, and *h*^2^ in the individual and combined environments, are shown in Supplementary Table 1. Our results showed that the plant heights ranged from 55.0 to 164.0 cm in the E1 and E3 environments, respectively. Moreover, the average heights among the environments varied from 99.58 ± 0.55 to 100.91 ± 0.55 cm in E1 and E3, respectively. In the combined environment, the CV was 17.23% and varied from 17.21 to 17.27% in E2/E3 and E1, respectively. In addition, the kurtosis and skewness were 0.42 and 0.26, respectively, in the combined environment (Supplementary Table 1). The broad-sense heritability (*h*^2^) in the combined environment was 0.95. Our results revealed a significant effect (*P* < 0.0001) of the soybean accessions (*G*) and environment (*E*) on plant height, whereas the genotype × environment interaction (*G* × *E*) was non-significant (Table 1).

**Table 1:** Analysis of variance (ANOVA) for plant height in the combined environments

Source	DF	SS	MS	*F*-value	P value (Prob > *F*)
Genotype (*G*)	195	759,377	3894	80.45	< 0.0001
Environment (*E*)	2	884	442	9.13	< 0.0001
*G* × *E*	390	2123	5	0.11	1.000
Error	2352	113,851	48		

*DF*, degrees of freedom; *SS*, sum of squares; *MS*, mean sum of squares; *E*, environment; *Prob, *probalitity; *P *value< 0.0001*, *significant; *P *value1.000, nonsignificant

### Marker quality control, population structure, and linkage disequilibrium analysis

**Fig. 1:**
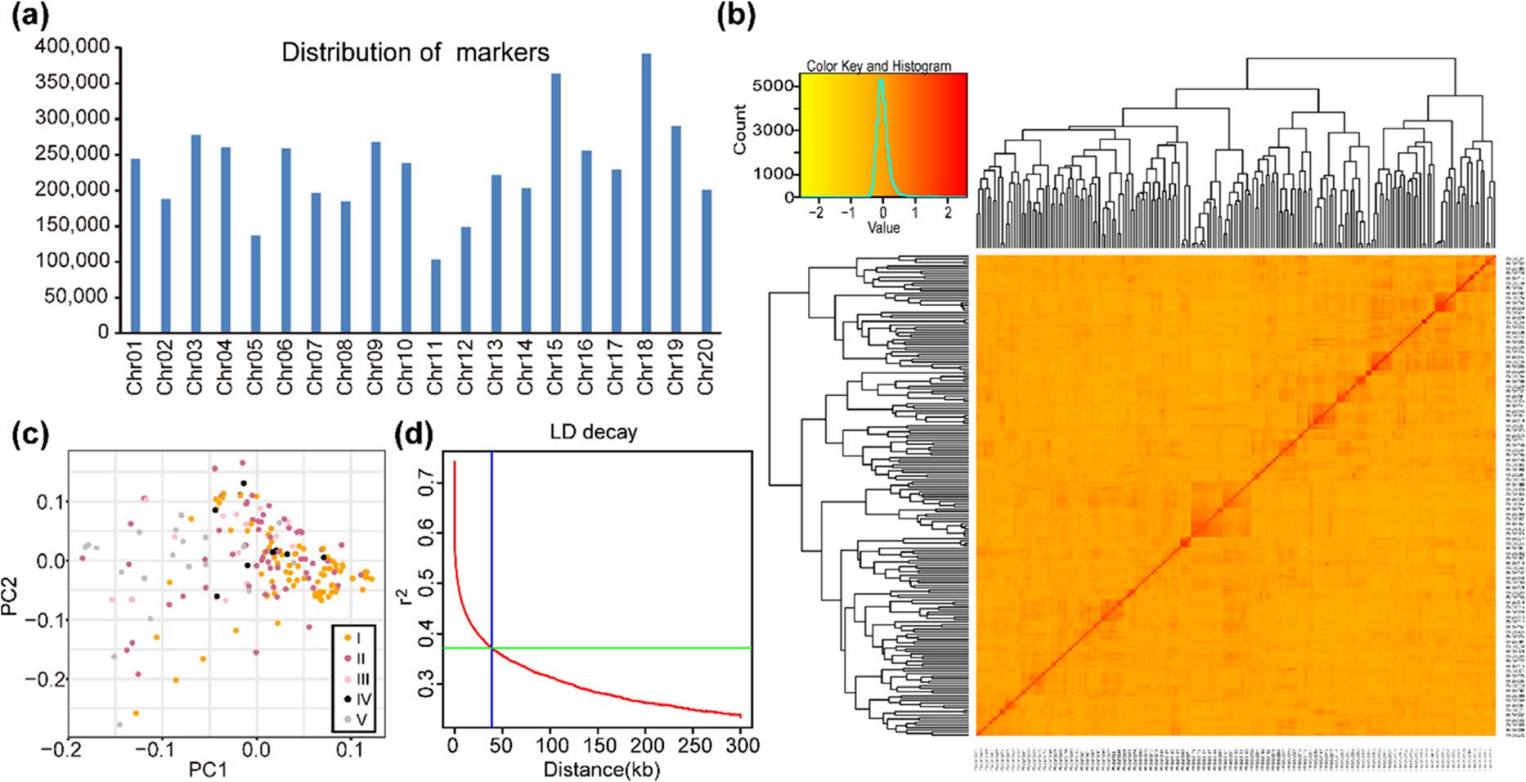
Marker distribution, kinship plot, population structure, and whole-genome LD decay plot of 196 soybean accessions collected from five accumulated temperature zones of northeastern China. **a** Genome-wide distribution of 4,665,814 SNP markers that are used for GWAS and are distributed across all soybean chromosomes. **b** Relationship of 196 soybean accessions depicted by a kinship plot. **c** Analysis of population structure for 196 soybean accessions collected from the five accumulated temperature zones, and in each zone the temperature remains constant. **d** LD decay plot of 196 soybean cultivars using 4,665,814 SNP markers. The LD decay fitted with a smoothing spline regression model is represented by the red curve line. The blue vertical line intersection with the horizontal green line represents the half-decay of LD (*r*.^2^ = 0.37), and the genetic distance at this point corresponds to LD decay distance (38.9 kb)

The resequencing of the 196 soybean accessions was carried out using the Illumina HiSeq platform, generating a total of 21.6G paired-end reads of 150 bp in length and 3.2 trillion bases, with an average coverage depth of 16 × . The raw paired-end resequencing reads were first cleaned by removing reads with adaptors as well as low-quality reads and reads with “N”s. After mapping against the soybean Williams 82 (*Wm82.a2.v1*) reference genome, we identified totals of 5,622,833 SNPs and 756,151 InDels. We performed the quality control analysis by removing the SNPs with MAF values of 0.05 and missing genotype frequencies at 0.10 among the 196 soybean accessions, and finally retained the 4,665,814 SNPs for further analysis and investigation. These SNP markers were present in all 20 soybean chromosomes, with the lowest number of 103,688 found on Chr.11 and the highest number of 392,184 found on Chr.18 (Fig. 1a; Table 2). The lowest and highest SNP densities of 2982.11 SNPs/Mb and 7029.75 SNPs/Mb were found on Chr.11 and Chr.15, respectively (Table 2; Supplementary Fig. 1). Based on the 4,665,814 polymorphic SNPs used to generate the heatmaps and dendrograms of the kinship matrix in the soybean panel, it was revealed that the genotypes showed no clear clustering behaviors (Fig. 1b). Moreover, the population structure analysis also showed no distinct structures with continuous distributions (Fig. 1c; Supplementary Table 2).

**Table 2:** Distribution of all the SNPs used for GWAS across the soybean chromosomes

Chromosome	Length (bp)	Length (Mb)	Numbers of SNPs	Inter-marker distance (bp)	Density (SNPs/Mb)
Chr01	56,831,624	56.83	243,969	232.95	4292.96
Chr02	48,577,505	48.58	188,500	257.71	3880.20
Chr03	45,779,781	45.78	277,739	164.83	6066.82
Chr04	52,389,146	52.39	260,152	201.38	4965.68
Chr05	42,234,498	42.23	136,674	309.02	3236.42
Chr06	51,416,486	51.42	259,575	198.08	5048.13
Chr07	44,630,646	44.63	196,744	226.85	4408.34
Chr08	47,837,940	47.84	185,415	258.00	3875.73
Chr09	50,189,764	50.19	267,859	187.37	5336.90
Chr10	51,566,898	51.57	238,839	215.91	4631.36
Chr11	34,766,867	34.77	103,688	335.30	2982.11
Chr12	40,091,314	40.09	149,078	268.93	3718.58
Chr13	45,874,162	45.87	221,482	207.12	4828.47
Chr14	49,042,192	49.04	203,567	240.91	4151.04
Chr15	51,756,343	51.76	363,860	142.24	7029.75
Chr16	37,887,014	37.89	256,272	147.84	6763.58
Chr17	41,641,366	41.64	229,537	181.41	5512.42
Chr18	58,018,742	58.02	392,184	147.94	6759.46
Chr19	50,746,916	50.75	289,978	175.00	5713.85
Chr20	47,904,181	47.90	200,702	238.68	4190.02
Total	949,183,385	949.19	4,665,814	216.78	4869.59

The LD characteristics of the 196 soybean accessions were also represented graphically and are presented in Fig. 1d. The average *r*^2^ value across the genome was 0.30, and the LD decay started at 0.74 and reached half-decay at 0.37. At the genomic distance of 38.9 kb, the intersection of the half-decay occurred with the LD decay curve and represents the critical genome-wide distance required to identify the linkage. Therefore, all the significant SNPs linked with plant height in this physical interval were regarded as QTLs.

### GWAS analysis for plant height

**Fig. 2:**
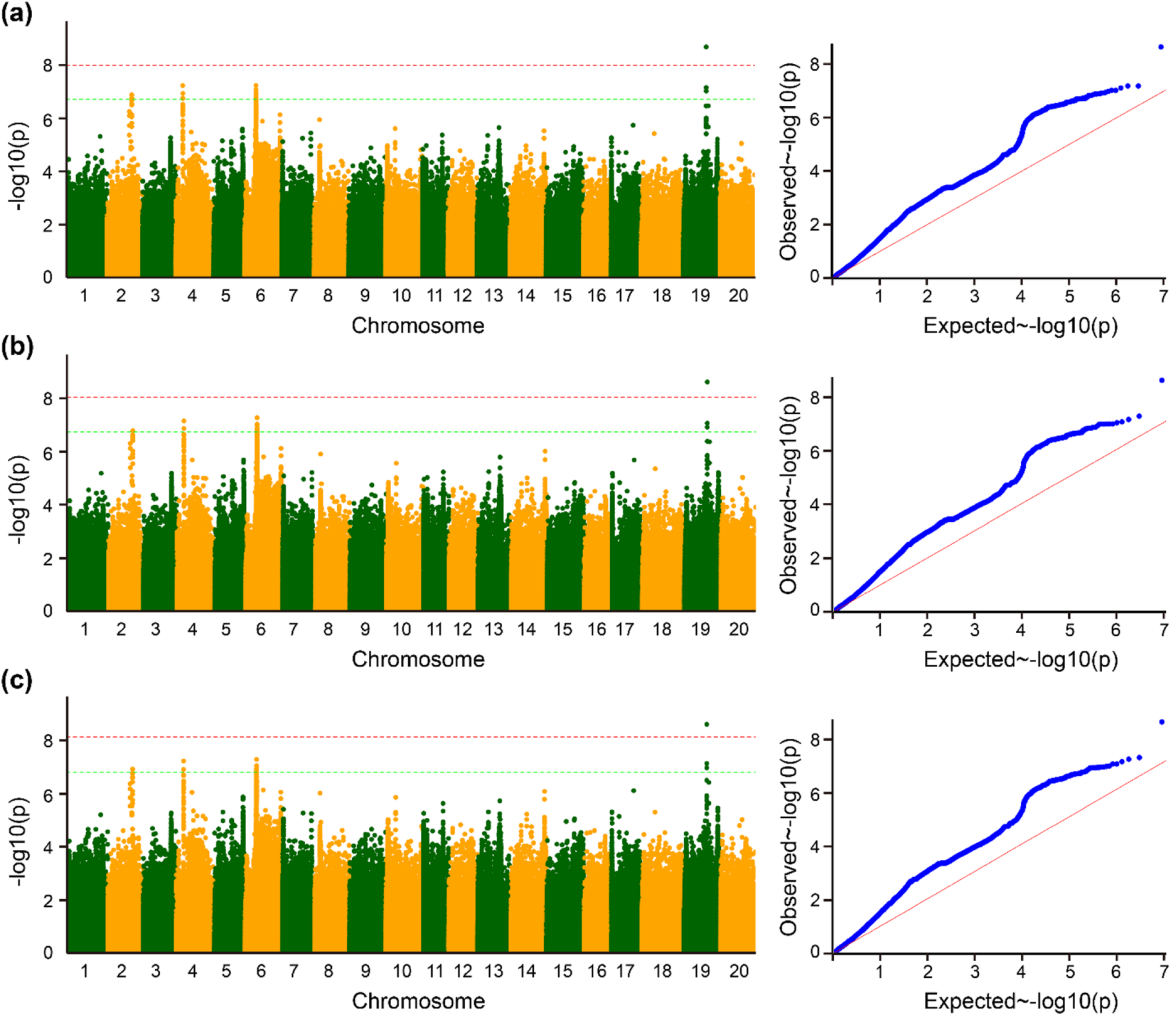
Genome-wide association study (GWAS) of plant height across three environments in soybean. The Manhattan plot and quantile–quantile plot of the GWAS results for plant height across three environments: **a** Jiamusi_2017 (E1), **b** Jiamusi_2018 (E2), **c** Jiamusi_2019 (E3). The green line represents the threshold level of significance (− log_10_*P* > 6.67), and the red line represents the threshold of − log10 (*p* value) after correction for the passing false-discovery rate (FDR), and the soybean chromosomes are represented by the numbers on the *X*-axis

Our study identified a total of 33 SNPs significantly linked with plant height across three different environments (Fig. 2; Table 3). These significant SNPs were distributed on four different chromosomes, viz., Chr.02, Chr.04, Chr.06, and Chr.19. Among these SNPs, the highest number of 18 SNPs was identified on Chr.06, followed by 9 SNPs on Chr.04 and 3 SNPs each on Chr.02 and Chr.19. Moreover, out of the 33 significant SNPs found to be associated with plant height, 23 were consistently detected in two or more environments. The significant association of these 23 SNPs across the different years of study in the Jiamusi location suggests that these significant SNPs are stable marker‒trait associations (MTAs). The remaining 10 significant SNPs were detected only in environment E1. Hence, most of the significant SNPs detected were stable MTAs. This suggests that environmental variables show consistency throughout the years of study in the Jiamusi location.

**Table 3:** Significant SNP markers associated with plant height across three environments (E1, E2, and E3)

S. no	Marker	Environment	Chr	Position	*P*_value
1	S02_33773119	E1	2	33,773,119	1.91E − 07
2	S02_33776150	E1, E3, E3	2	33,776,150	1.43E − 07
3	S02_33776153	E1, E2, E3	2	33,776,153	1.43E − 07
4	S04_9734981	E1, E2, E3	4	9,734,981	6.43E − 08
5	S04_9735016	E1, E2, E3	4	9,735,016	1.26E − 07
6	S04_9735831	E1	4	9,735,831	1.97E − 07
7	S04_9735844	E1	4	9,735,844	1.97E − 07
8	S04_9735858	E1	4	9,735,858	1.97E − 07
9	S04_9738503	E1	4	9,738,503	1.89E − 07
10	S04_9738519	E1	4	9,738,519	1.89E − 07
11	S04_9740763	E1	4	9,740,763	1.95E − 07
12	S04_9740805	E1	4	9,740,805	1.95E − 07
13	S06_15911596	E1, E2	6	15,911,596	1.63E − 07
14	S06_15944709	E1, E2, E3	6	15,944,709	1.13E − 07
15	S06_15947237	E1, E2, E3	6	15,947,237	1.36E − 07
16	S06_15947254	E1, E2, E3	6	15,947,254	1.85E − 07
17	S06_15950577	E1	6	15,950,577	2.12E − 07
18	S06_15953906	E1, E2, E3	6	15,953,906	1.63E − 07
19	S06_15955748	E1, E2, E3	6	15,955,748	6.43E − 08
20	S06_15956887	E1, E2, E3	6	15,956,887	1.42E − 07
21	S06_15956890	E1	6	15,956,890	2.05E − 07
22	S06_15959421	E1, E2	6	15,959,421	1.85E − 07
23	S06_15959429	E1, E2	6	15,959,429	1.85E − 07
24	S06_15959441	E1, E2	6	15,959,441	1.85E − 07
25	S06_15959554	E1, E2, E3	6	15,959,554	1.29E − 07
26	S06_15959556	E1, E2, E3	6	15,959,556	1.29E − 07
27	S06_15959559	E1, E2, E3	6	15,959,559	1.22E − 07
28	S06_15959658	E1, E2, E3	6	15,959,658	9.30E − 08
29	S06_15959681	E1, E2, E3	6	15,959,681	9.30E − 08
30	S06_15967346	E1, E2, E3	6	15,967,346	1.16E − 07
31	S19_31514448	E1, E2, E3	19	31,514,448	1.03E − 07
32	S19_31514472	E1, E2, E3	19	31,514,472	2.22E − 09
33	S19_31514500	E1, E2, E3	19	31,514,500	7.59E − 08

*E1*, Jiamusi_2017; *E2*, Jiamusi_2018; *E3*, Jiamusi_2019

Based on the LD decay results, the genomic regions around the 3, 9, 18, and 3 significant SNPs identified on Chr.02, Chr.04, Chr.06, and Chr.19, respectively, covered the physical intervals of 3.03 kb, 5.82 kb, 55.75 kb, and 0.05 kb, respectively, on the soybean genome, and these genomic regions all fell within the ± 38.9 kb flanking physical interval. Hence, they could be considered four stable QTLs regulating the soybean plant height, viz., *qPH2*, *qPH4*, *qPH6*, and *qPH19* (Table 4). These QTL/genomic regions showed consistency across the years of study in the Jiamusi location, suggesting the stable nature of these QTLs governing the soybean plant height.

**Table 4:** Quantitative trait loci consistently linked with plant height across all three environments (E1, E2 and E3)

QTL	Chromosome	Representative SNP^a^	Position (bp)	Number of significant SNPs	Environments	Related QTL	References
*qPH2*	Chr.02	S02_33776150	33,776,150	03	E1, E2, and E3	*Plant height 35–5*	Rossi et al. (2013)
*qPH4*	Chr.04	S04_9735858	9,735,858	09	E1, E2, and E3	No related QTL	Not available
*qPH6*	Chr.06	S06_15956887	15,956,887	18	E1, E2, and E3	No related QTL	Not available
*qPH19*	Chr.19	S19_31514472	31,514,472	03	E1, E2, and E3	*Plant height 22–1*	Li et al. (2008a, b)

^a^The representative SNP with the minimum *P*-value

*E1*, Jiamusi_2017; *E2*, Jiamusi_2018; *E3*, Jiamusi_2019

### Superior haplotypes for plant height

The significant SNPs linked to the same trait falling within the LD decay range of ± 38.9 kb form the haplotype blocks. Our results showed that all the significant SNPs identified on Chr.02, Chr.04, Chr.06, and Chr.19 were present within the LD decay range of ± 38.9 kb, and these SNPs thus form four haplotype blocks on their respective chromosomes (Supplementary Table 3). For example, the 3 significant SNPs within 3.03-kb physical regions on Chr.02 form the Hap-2 haplotype block. On Chr.04, the 9 significant SNPs present within the 5.82-kb physical interval form the Hap-4 haplotype block. Similarly, the 18 significant SNPs present within the 55.75-kb range form the Hap-6 haplotype block on Chr.06. Moreover, the 3 significant SNPs on Chr.19 in the physical interval of 0.05 kb form the Hap-19 haplotype block.

**Fig. 3:**
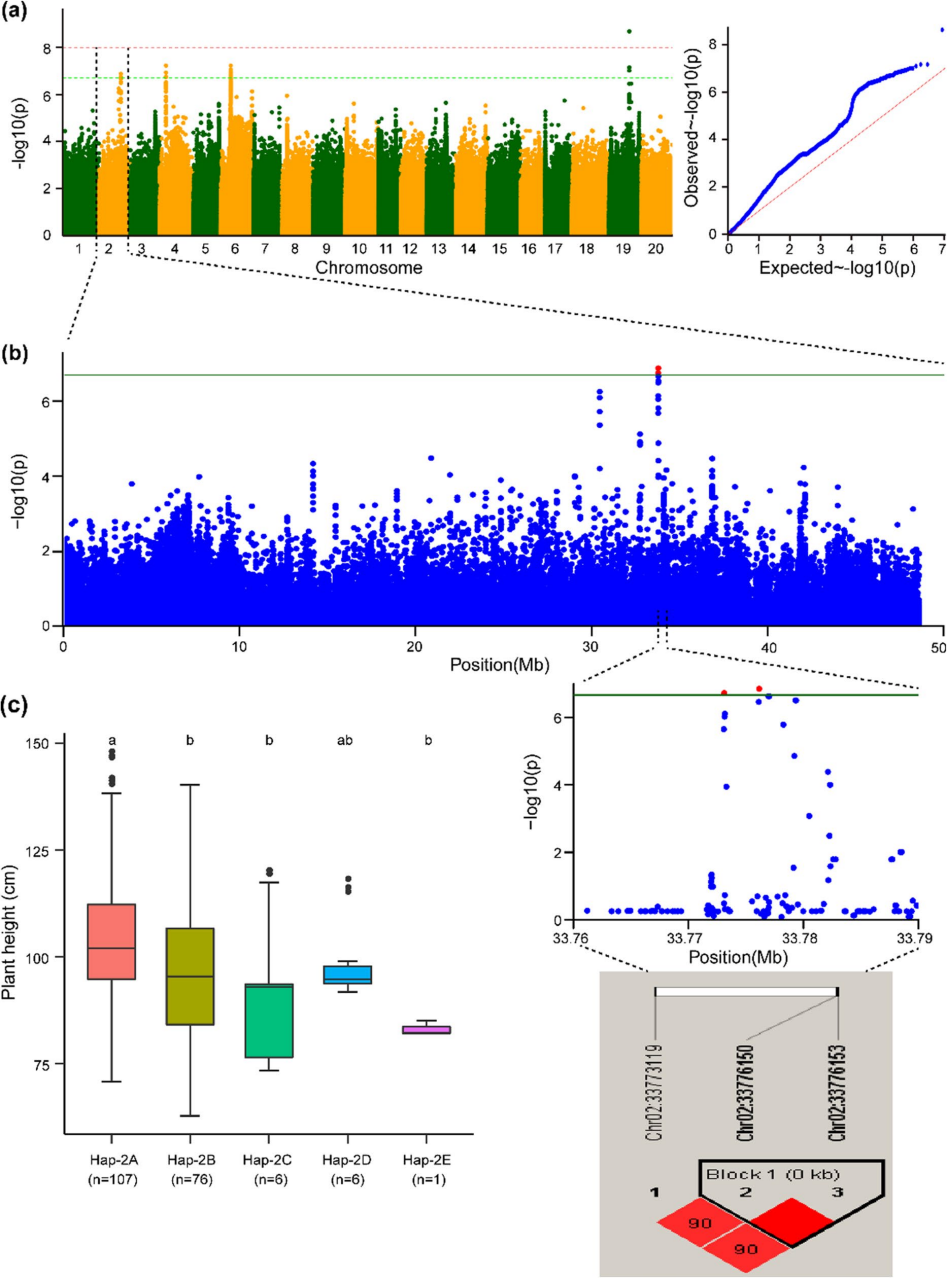
Haplotype alleles detected in the haplotype block (Hap-2) on Chr.02 regulate varied phenotypes of plant height in soybean. **a** Manhattan and quantile–quantile plots from the combined environments for the GWAS of plant height. **b** GWAS signal results for plant height on Chr.02 and pairwise LD analysis. For the significant variants (*P* < 2.14 × 10^−7^), a pairwise LD diagram is displayed. **c** Predicted plant height values from combined environments are shown by the haplotype boxplot. Tukey’s HSD test at *P* < 0.05 was used to group the genotypes and conduct pairwise comparisons. Nonsignificant differences in plant height are represented by common letters above the boxes. On the horizontal axis, “*n*” represents the accession number in each subgroup

**Fig. 4:**
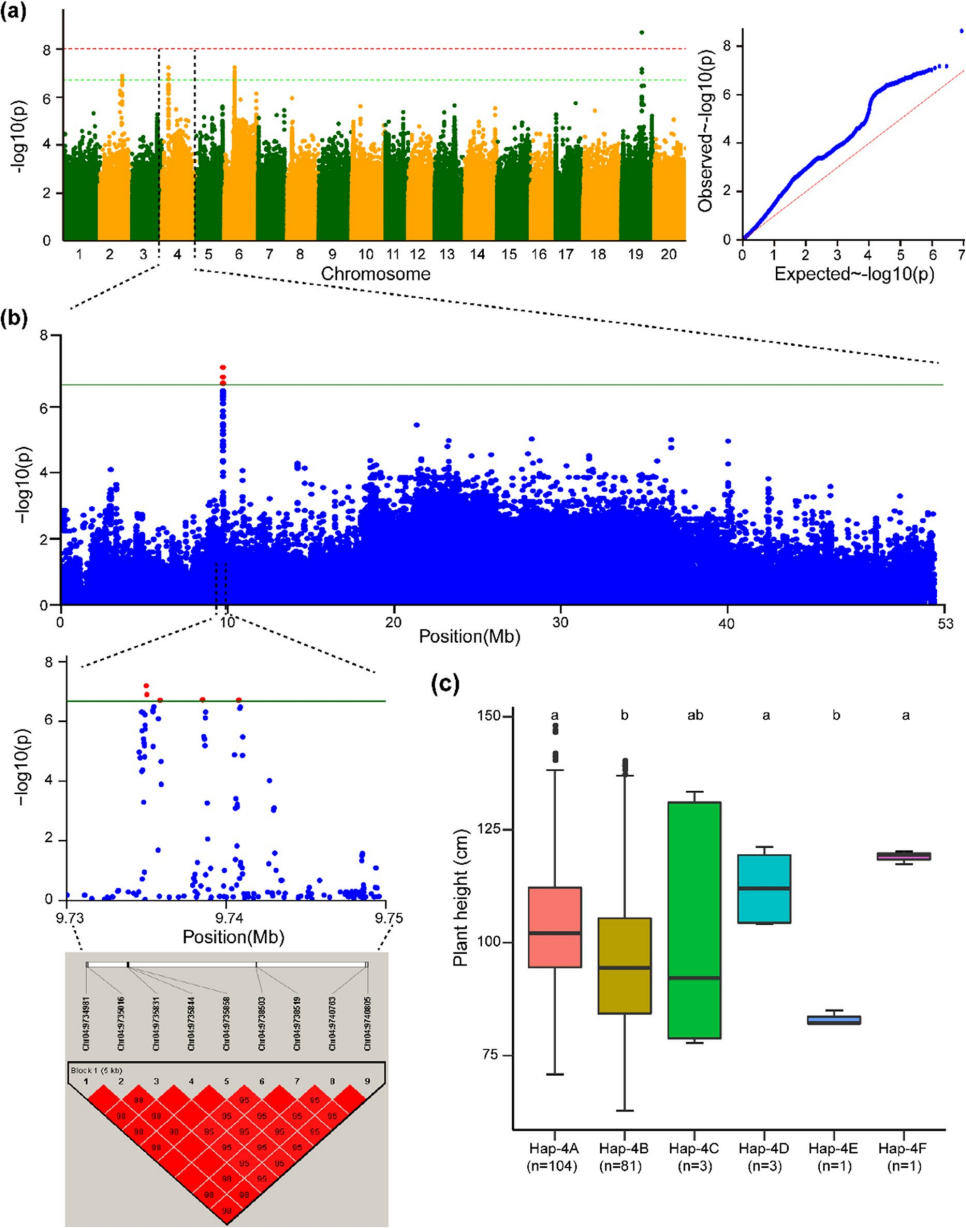
Haplotype alleles detected in the haplotype block (Hap-4) on Chr.04 regulate varied phenotypes of plant height in soybean. **a** Manhattan and quantile–quantile plots from e combined environments for the GWAS of plant height. **b** GWAS signal results for plant height on Chr.04 and pairwise LD analysis. For the significant variants (*P* < 2.14 × 10^−7^), a pairwise LD diagram is displayed. **c** Predicted plant height values from combined environments are shown by the haplotype boxplot. Tukey’s HSD test at *P* < 0.05 was used to group the genotypes and conduct pairwise comparisons. Nonsignificant differences inr plant height are represented by common letters above the boxes. On the horizontal axis, “*n*” represents the accession number in each subgroup

**Fig. 5:**
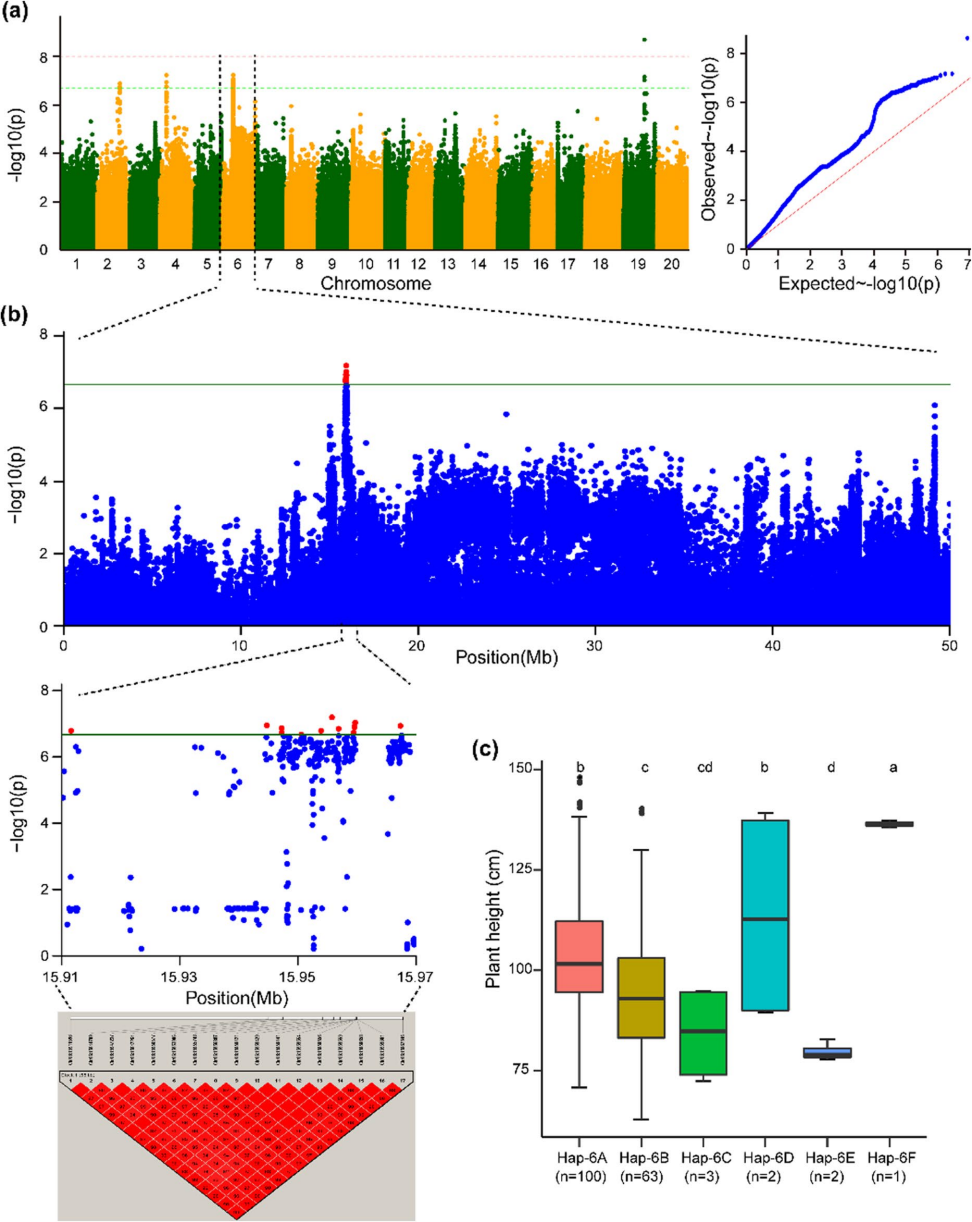
Haplotype alleles detected in the haplotype block (Hap-6) on Chr.06 regulate varied phenotypes of plant height in soybean. **a** Manhattan and quantile–quantile plots from combined environments for the GWAS of plant height. **b** GWAS signal results for plant height on Chr.06 and pairwise LD analysis. For the significant variants (*P* < 2.14 × 10^−7^), a pairwise LD diagram is displayed. **c** Predicted plant height values from combined environments are shown by the haplotype boxplot. Tukey’s HSD test at *P* < 0.05 was used to group the genotypes and conduct pairwise comparisons. Nonsignificant differences in plant height are represented by common letters above the boxes. On the horizontal axis, “*n*” represents the accession number in each subgroup

**Fig. 6:**
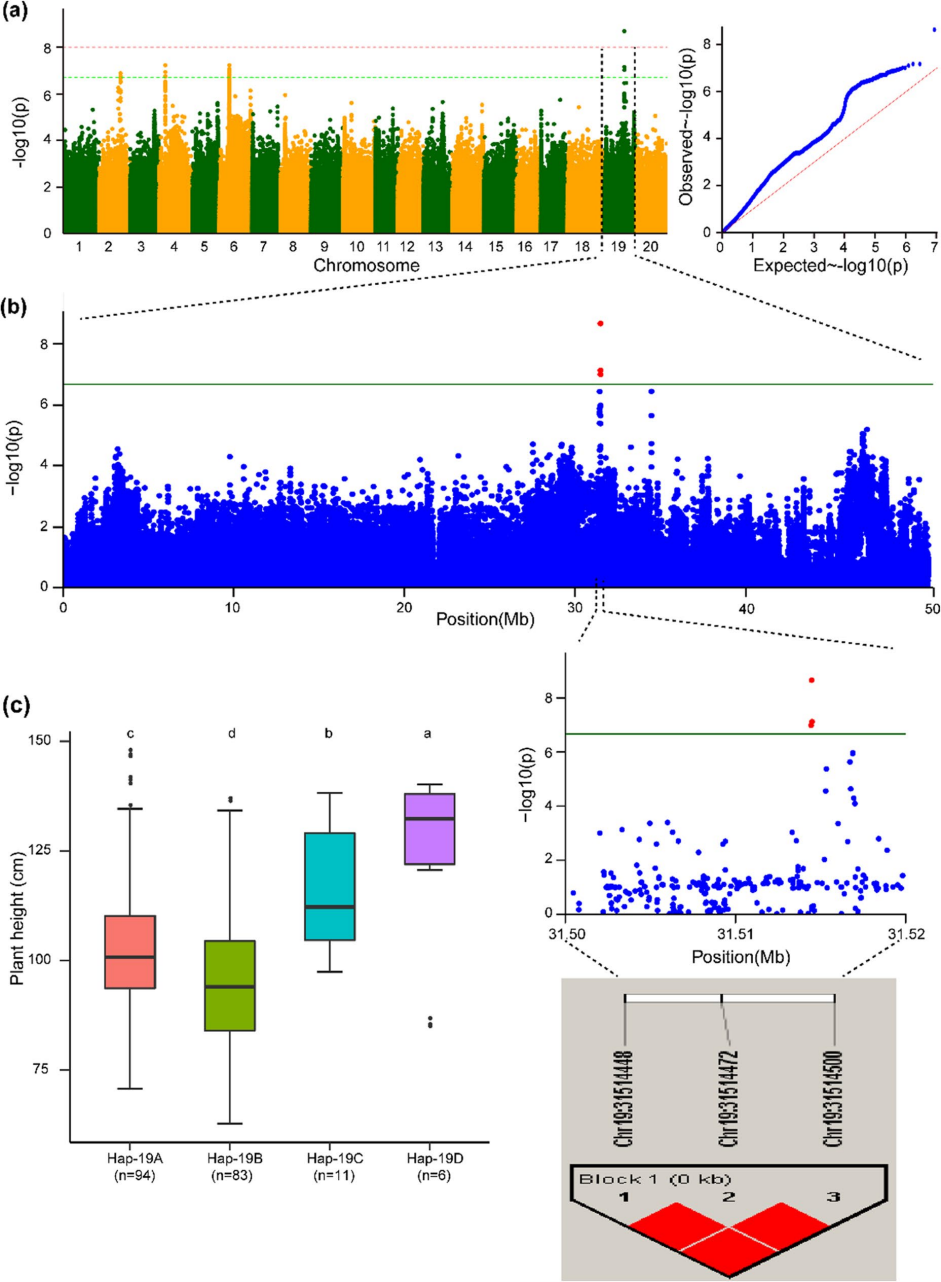
Haplotype alleles detected in the haplotype block (Hap-19) on Chr.19 regulate varied phenotypes of plant height in soybean. **a** Manhattan and quantile–quantile plots from combined environment for the GWAS of plant height. **b** GWAS signal results for plant height on Chr.19 and pairwise LD analysis. For the significant variants (*P* < 2.14 × 10^−7^), a pairwise LD diagram is displayed. **c** Predicted plant height values from combined environments are represented by the haplotype boxplot. Tukey’s HSD test at *P* < 0.05 was used to group the genotypes and conduct pairwise comparisons. Nonsignificant differences in plant height are represented by common letters above the boxes. On the horizontal axis, “*n*” represents the accession number in each subgroup

The haplotype block of Chr.02 possesses five haplotype alleles across the 196 soybean accessions, and these alleles can be used to group the soybean accessions into five groups. Significant differences were observed among these five haplotypes, viz., Hap-2A, Hap-2B, Hap-2C, Hap-2D, and Hap-2E, with regard to their governing behavior of the plant height phenotype, and these haplotypes represent 107, 76, 6, 6, and 1 soybean genotypes, respectively (Fig. 3c). Hap-2E regulates the dwarf phenotype, Hap-2A governs the tallest phenotype, and the remaining three haplotypes (Hap-2B, Hap-2C, and Hap-2D) govern the intermediate and semidwarf plant height phenotypes. The haplotype block Hap-4 on Chr.04 possesses six haplotypes across 196 accessions of the natural soybean population (Fig. 4c). Notably, these haplotypes classify the accessions of the soybean population into six groups. The plant height of the accessions varied significantly among the six haplotype groups. For instance, Hap-4A formed the largest group (*n* = 104), followed by Hap-4B (*n* = 81), Hap-4C (*n* = 3), Hap-4D (*n* = 3), Hap-4E (*n* = 1), and Hap-4F (*n* = 1) (Fig. 4c). However, the soybean accessions with Hap-4F appeared to be the tallest across all environments, those with Hap-4E had a dwarf phenotype, and accessions with Hap-4A, Hap-4B, Hap-4C, and Hap-4D possessed intermediate plant heights (Fig. 4c). Similarly, the haplotype block Hap-6 identified on Chr.06 possessed six haplotype alleles across the 196 soybean accessions (Fig. 5c). Significant differences were observed among the six haplotype alleles, and Hap-6A, Hap-6B, Hap-6C, Hap-6D, Hap-6E, and Hap-6F were present in 100, 63, 3, 2, 2, and 1 soybean accessions, respectively (Fig. 5c). Hap-6F governs the tallest plant height, and Hap-6E controls the dwarf plant height phenotype, whereas the remaining haplotype alleles present within Hap-6 regulate the plant heights within the range lying between these two phenotypes. Moreover, the haplotype block Hap-19 identified on Chr.19 possesses four haplotype alleles, viz., Hap-19A, Hap-19B, Hap-19C, and Hap-19D, and these haplotype alleles were present in 94, 83, 11, and 6 soybean accessions, respectively (Fig. 6c). The haplotype alleles Hap-19D and Hap-19B control the tallest height and dwarf phenotype, respectively, and the remaining two haplotype alleles regulate the plant height in the intermediate range. In conclusion, the haplotype alleles identified within the Hap-2, Hap-4, Hap-6, and Hap-19 blocks regulate the different plant height phenotypes ranging from dwarf-semidwarf-intermediate-tall.

### Candidate gene detection under haplotype blocks

Candidate gene identification was performed within the physical interval of four haplotype blocks, viz., Hap-2, Hap-4, Hap-6, and Hap19. To this end, we identified two, two, four, and one candidate genes within the haplotype blocks of Hap-2, Hap-4, Hap-6, and Hap19, respectively (Table 5). Moreover, we also retrieved the free RNA-seq data available at SoyBase (https://www.soybase.org/), and the RNA-seq data of seven genes out of a total of nine identified genes were available based on the online dataset at SoyBase (https://www.soybase.org/). These RNA-seq data represent fourteen soybean tissues at various growth and development stages (Supplementary Table 4). A heatmap was used to represent the expression patterns of these genes based on the RNA-seq data (Supplementary Fig. 2). The gene annotation analysis showed that functional annotations were available for five genes out of a total of nine genes. Hence, combining the locations of the genes within the haplotype blocks together with the in silico analysis results of the gene expression data and gene annotations, these nine candidate genes are considered possible genes regulating plant height in soybean. However, further functional validation is needed to confirm their true function and deployment capacity in soybean breeding programs.

**Table 5:** Potential candidate genes underlying four haplotype blocks namely, Hap-2, Hap-4, Hap-6, and Hap-19, and their gene annotation

S. no	Gene ID (*Wm82.a2.v1*)	Biological function
1	*Glyma.02g186300*	Positive regulation of translation; protein phosphorylation; regulation of DNA replication; regulation of G2/M transition of mitotic cell cycle
2	*Glyma.02g186400*	NA
3	*Glyma.04g103900*	Cellular response to salt stress; cytokinin-mediated signaling pathway; ethylene-mediated signaling pathway; red or far-red light signaling pathway; regulation of cell differentiation; regulation of transcription, DNA-dependent; response to cold; response to light stimulus; response to osmotic stress; response to salt stress; response to water deprivation; response to wounding
4	*Glyma.04g104000*	NA
5	*Glyma.06g184100*	Spermidine biosynthetic process
6	*Glyma.06g184200*	Metabolic process; response to fructose stimulus; response to sucrose stimulus; trehalose biosynthetic process
7	*Glyma.06g184300*	NA
8	*Glyma.06g184400*	Acetyl-CoA metabolic process; auxin polar transport; brassinosteroid biosynthetic process; growth; microtubule nucleation; pattern specification process; plant-type cell wall biogenesis; plant-type cell wall organization; polysaccharide catabolic process; protein phosphorylation; regulation of cell size; regulation of meristem growth; root morphogenesis; starch metabolic process; sterol biosynthetic process; transmembrane receptor protein tyrosine kinase signaling pathway
9	*Glyma.19g088500*	Biological process

## Discussion

Plant height is a key trait regulating soybean plant types, which in turn affects the soybean yield and quality (Zhao et al. 2022). The development of a desirable plant type is a long-term goal of soybean breeders (Cheng et al. 2019). In this context, characterizations of soybean germplasm collections with regard to the plant type/height should be intensely performed to accelerate soybean improvement. Manipulating the soybean plant height to produce a desirable plant type is an important goal of soybean breeding programs (Cao et al. 2019). To this end, it is necessary to first elucidate the detailed genetic basis of the soybean plant height and deploy the identified plant height genes/QTLs for soybean improvement. In this regard, the present investigation was performed to detect the QTLs, superior haplotypes, and candidate genes regulating plant height in soybean by using a combined approach involving GWAS, haplotype, and candidate gene analyses.

In our study, we used 196 diverse soybean accessions collected from five accumulated temperature zones of north-eastern China to characterize plant height at the Jiamusi Experimental Farm in the three consecutive years of 2017, 2018, and 2019 representing the E1, E2, and E3 environments, respectively. The plant height showed significant genotypic variance among the soybean accessions, thus providing a foundation for assessing the potential to manipulate the plant height, as has been previously documented in other studies (Cao et al. 2019; Fang et al. 2020). Our results showed significant effects of the genotype (*G*) and environment (*E*) on plant height, but the *G* × *E* interactions were non-significant. The results can be explained using the three analyzed environments, viz., E1, E2, and E3, representing the same Jiamusi locations but in three consecutive years. Hence, the environmental variables remained almost constant across the years of study in the Jiamusi location, which in turn resulted in the consistent performance of the soybean genotypes affecting plant height across the environments. Moreover, the higher broad-sense heritability observed for plant height across environments indicated that the same phenotypic performance is achievable if plants are grown in the same environment. In agreement with our findings, many authors have previously reported similar results for plant height in the soybean germplasm collection (Xue et al. 2019; Cao et al. 2019; Bhat et al. 2022). However, the significant genotypic and environmental variances observed for plant height suggest a complex plant height inheritance pattern, which is in agreement with the findings of Lee et al. (2015).

Multiple studies have been performed to elucidate the genetic basis of plant height in soybean (Orf et al. 1999; Chapman et al. 2003; Zhang et al. 2004; Li et al. 2008a, b; Liu et al. 2013). Most of these studies have used low-density markers and traditional linkage mapping approaches; the demerits of their low-resolution gene mapping results have affected the successful deployment of the results in marker-assisted breeding programs (Bhat et al. 2021). In this context, the recent application of the GWAS analysis approach using high-density markers has emerged as a powerful and high-resolution method for identifying genes regulating the traits of interest (Alqudah et al. 2020). Earlier studies have documented that GWAS has great potential to unravel the genetic makeup of quantitative traits such as plant height at a higher accuracy than those obtained in previous studies (Cober and Morrison 2010; Tam et al. 2019). Thus, our study used this approach to determine the genetic architecture of the soybean plant height. A total of 33 SNPs were identified to be linked with plant height across all three environments, and these SNPs were distributed on four chromosomes, viz., Chr.02, Chr.04, Chr.06, and Chr.19. The distribution of significant SNPs on different chromosomes suggests the complex genetic basis of plant height in soybean. This result is similar to those previously reported by other authors studying the soybean plant height (Chang et al. 2018; Xue et al. 2019; Cao et al. 2019). Furthermore, the greatest number of significant SNPs (23 out of total 33) linked to PH was found to be consistent across the two or three environments. The three environments represent the three different years, viz., 2017, 2018, and 2019, in the same location, with the environmental variables remaining constant across the years. These findings suggest that although plant height is affected by the environment, the existence of constant environmental variables across multiple environments does not affect the plant height, which is in accordance with previous reports (Fang et al. 2020; Bhat et al. 2022).

Based on the LD decay results, all the SNPs identified on Chr.02, Chr.04, Chr.06, and Chr.19 fell within the ± 38.9 kb flanking physical interval, and hence were considered four QTLs, viz., *qPH2*, *qPH4*, *qPH6*, and *qPH19*, respectively. Previously, Rossi et al. (2013) identified a QTL on Chr.02 (*plant height 35–5*) linked with plant height in the physical interval of 31,234,716 and 34,993,207 bp; however, the physical interval of *qPH2* falls within this genomic region. Hence, *qPH2* could be the same as the *plant height 35–5* identified by Rossi et al. (2013). Moreover, *qPH19* is present within the physical interval of *plant height 22–1* (31,391,445–35,672,961) previously reported by Li et al. (2008a, b). However, *qPH4* and *qPH6* are novel QTLs, as no previously reported QTL falls within the physical interval of *qPH4* or *qPH6*. Our study also demonstrated that the physical intervals of *qPH2* and *qPH19* were substantially narrowed in the current study. *Plant height 35–5* was previously identified by using low-resolution approaches involving linkage mapping and low-density markers. The linkage mapping approach utilizes biparental mapping populations, which leads to reduced mapping resolution in the QTL detection results corresponding to the traits of interest (Kraakman et al. 2004). In this regard, the GWAS approach uses the natural diverse population and ancestral recombination events, thereby provides high-resolution gene mapping results. Hence, the identification of stable QTLs across multiple environments through GWAS critically allows their successful and efficient deployment in crop breeding programs for developing soybean plants with desirable plant types.

The potential of applying the GWAS approach in gene identification research targeting complex traits has been well-recognized by the research community. However, biallelic SNP markers have largely been used for GWAS analyses of soybean, resulting in the loss of superior and rare alleles regulating desirable phenotypes for crop traits (Bhat et al. 2021). In this regard, multiallelic haplotypes provide a solution to capture these superior and rare alleles as well as epistatic variations in crop traits (Luján Basile et al. 2019). Hence, haplotype analyses provide an enormous opportunity to exploit the maximum genetic variations in crop improvement. For example, Patil et al. (2016) identified multiple haplotypes underlying the salt tolerance gene *GmCHX1*, and among them, the maximum salt tolerance was provided by the haplotype allele *SV-2*. Similarly, superior haplotypes were identified for other traits in different crops, such as drought tolerance in pigeonpea (Sinha et al. 2020a, b) and grain quality traits (Wang et al. 2017). Haplotype variations across the crop germplasm are governed by recombination, mutation, and selection (Zaitlen et al. 2005). Hence, identifying haplotype variations and successfully deploying such variations in crop breeding programs are extremely important for harnessing the true potential of genetic diversity in crop improvements (Sinha et al. 2020a, b). In our study, we identified four haplotype blocks, viz. Hap-2, Hap-4, Hap-6, and Hap-19, on Chr.02, Chr.04, Chr.06, and Chr.19, respectively. Hap-2, Hap-4, Hap-6, and Hap-19 possess five, six, six, and four haplotype alleles, respectively. Our results showed that the alleles corresponding to each haplotype regulate the different plant height phenotypes, and significant differences were observed among the haplotype alleles underlying each haplotype block. The phenotypes regulated by the haplotype alleles underlying these four haplotype blocks varied from dwarf to tall. The haplotype alleles of each block govern the different plant height phenotypes, thus providing the opportunity to modify the soybean plant height in multiple ways as per the breeder’s requirements. The deployment of these haplotypes in soybean breeding programs will allow us to produce soybean cultivars with the desired plant heights and types, which in turn will have a great impact on the soybean yield and quality.

The identification of candidate genes for crop traits and their proper functional validation as well as deployment in crop breeding programs are the ultimate goals of researchers (Ganie and Ahammed 2021; Ganie et al. 2021). In the past, negligible efforts have been made to detect genes governing plant height in soybean (as reviewed by Bhat et al. (2022), and limited genes governing plant height have been characterized (Liu et al. 2010; Ping et al. 2014; Zhang et al. 2018). In our study, an in silico analysis detected nine candidate genes underlying Hap-2, Hap-4, Hap-6, and Hap-19, and these genes were defined as possible candidates modulating plant height in soybean. It is well known that the gene functions related to cell division, mitosis, photosynthesis, growth hormones, cell elongation, or other functions related to vegetative growth directly or indirectly govern plant heights. Hence, among the nine identified genes, the functions of four genes, viz., *Glyma.02g186300*, *Glyma.04g103900*, *Glyma.06g184200*, and *Glyma.06g184400*, were found to be related to cell division, mitosis, photosynthesis, growth hormones, cell elongation, and vegetative growth, revealing their function in affecting plant heights (Table 5). Shan et al. (2021) documented that cell division and elongation promoted by plant growth hormones such as gibberellins (GAs) increase stem elongation and height in soybean. Moreover, cytokinin and auxin have been widely reported to control cell division (Yang et al. 2021 and Takatsuka and Umeda 2014). Auxin promotes cell division and meristem maintenance, thus playing an important role in the establishment of cellular patterning (Perrot-Rechenmann 2010). Moreover, the *Glyma.06g184100* function is related to the spermidine biosynthetic process, and previous studies have documented that spermidine activates the target of rapamycin (TOR), which in turn stimulates the growth of maize and *Arabidopsis* plants (Salazar-Díaz et al. 2021). TOR acts as a central nutrient sensor, especially for N, thus serving as a key regulator of plant development and growth (Salazar-Díaz et al. 2021). The gene function annotations of the other four candidate genes, viz., *Glyma.02g186400*, *Glyma.04g104000*, *Glyma.06g184300*, and *Glyma.19g088500*, are not known. Thus, to deploy these genes in molecular breeding, it is first necessary to verify the functions of these genes by using knockout or overexpression experiments, after which they can be directly used in the breeding of soybean crops with desirable plant heights.

## Conclusion

In the current study, we combined GWAS, haplotype, and candidate gene analyses to unravel the genetic basis of the soybean plant height. Totals of 33 significant SNPs and four stable QTLs, viz., *qPH2*, *qPH4*, *qPH6*, and *qPH19*, were found to be associated with plant height. Out of these four QTLs, two were novel, *qPH4* and *qPH6*, and the remaining two, viz., *qPH2* and *qPH19*, were previously reported. In addition, four haplotype blocks, viz., Hap-2, Hap-4, Hap-6, and Hap-19, were detected on Chr.02, Chr.04, Chr.06, and Chr.19, respectively. The number of haplotype alleles underlying each haplotype block varied from four to six, and significant differences were detected among the haplotype alleles of each block for plant height phenotypes ranging from dwarf to extra-tall. Moreover, a total of nine candidate genes were identified within haplotype blocks and considered possible genes regulating plant height. The QTLs and superior haplotype alleles identified herein can serve as important resources for breeding soybean crops with desirable plant heights. Moreover, following their proper validation through knockout or overexpression studies, the candidate genes identified herein can also be deployed in soybean breeding programs. Hence, the QTLs, haplotypes, and candidate genes identified in the present study will serve as important genetic resources for developing soybean varieties possessing desirable plant types.

## Supplementary Information

Below is the link to the electronic supplementary material.

Supplementary file1 (DOCX 338 KB)

Supplementary file2 (DOCX 15 KB)

Supplementary file3 (XLSX 13 KB)

Supplementary file4 (XLSX 16 KB)

Supplementary file5 (XLSX 10 KB)

## Data Availability

Resequencing data generated in this study has been deposited in the VCF format at the Genome Variation Map (GVM) database in BIG Data Center (http://bigd.big.ac.cn/gvm) under the accession number GVM00044.
