# Supplementary material for: Genome-wide survey identified superior and rare haplotypes for plant height in the north-eastern soybean germplasm of China
Source: Mol Breed. 2023 Mar 20;43(4):22. doi: 10.1007/s11032-023-01363-7 (PMC10248691; doi:10.1007/s11032-023-01363-7)
Supplement: Supplementary file 1 — Supplementary file1 (DOCX 338 KB) [file 11032_2023_1363_MOESM1_ESM.docx]

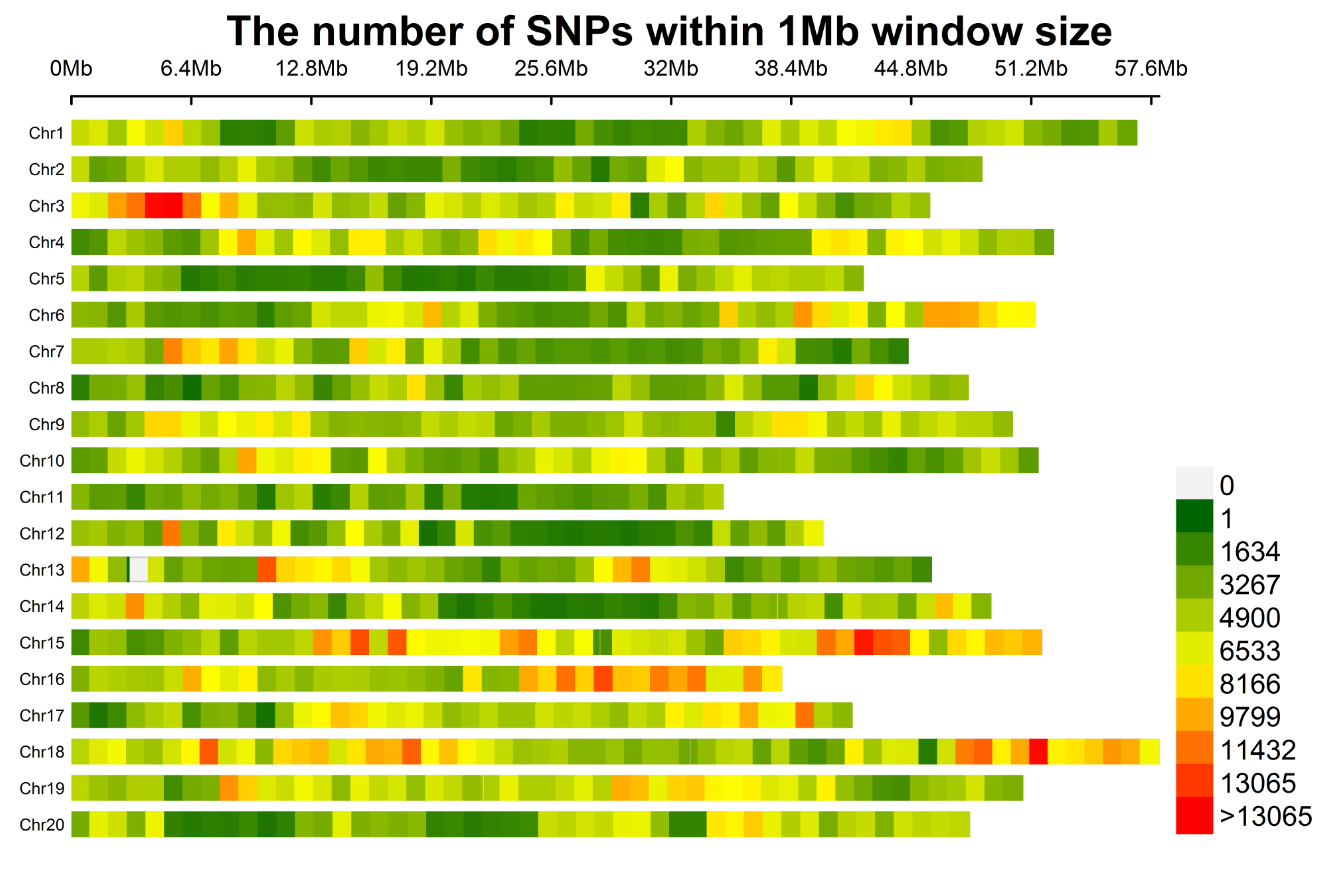


**Supplementary Fig. 1** This diagram shows the presence of the 4,665,814 SNPs across twenty soybean chromosomes. Length of chromosomes (Mb) is represented by the horizontal axis; chromosome number is denoted by the vertical axis, and SNP density is depicted by the different colors (number of SNPs per window).


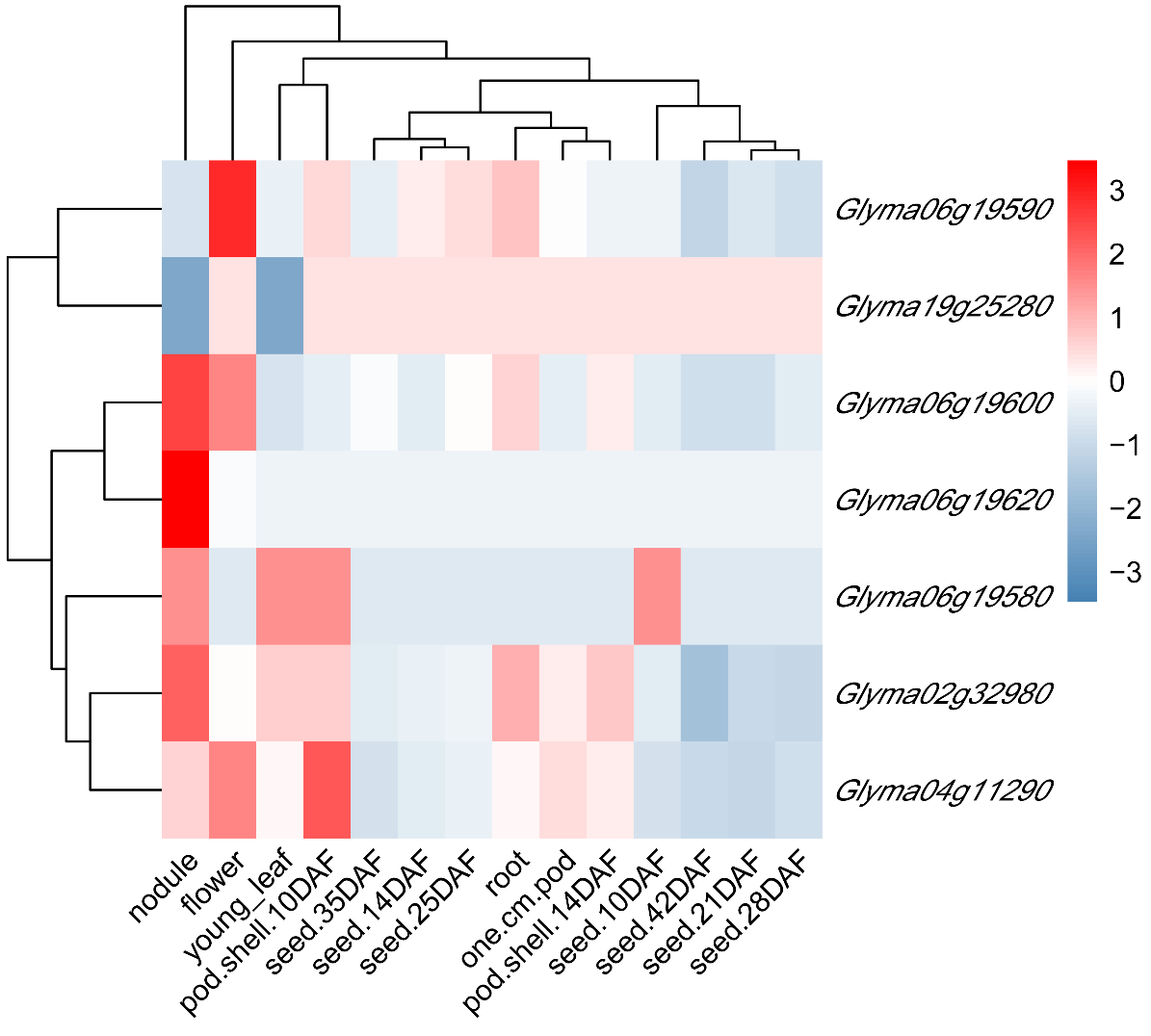


**Supplementary Fig. 2** Heat map showed gene expression profile of seven candidate genes identified within the four haplotype blocks; that represents the one, one, four and one genes on Chr.02, Chr.04, Chr.06 and Chr.19. This RNA-seq data was retrieved from the online dataset at SoyBase, and it represents the growth and development stages of soybean. DAF (days to flowering); cm (centimeter).
