# Supplementary material for: Genome-wide survey identified superior and rare haplotypes for plant height in the north-eastern soybean germplasm of China
Source: Mol Breed. 2023 Mar 20;43(4):22. doi: 10.1007/s11032-023-01363-7 (PMC10248691; doi:10.1007/s11032-023-01363-7)
Supplement: Supplementary file 2 — Supplementary file2 (DOCX 15 KB) [file 11032_2023_1363_MOESM2_ESM.docx]

**Supplementary Table 1** Phenotypic analysis viz., range (minimum and maximum values), mean, standard deviation (SD), coefficient of variation (CV), skewness, kurtosis and broad-sense heritability (*h^2^*) for plant height evaluated in 196 diverse soybean cultivars across three environments.

| Environment | Minimum | Maximum | Mean±SE | SD | CV% | Skewness | Kurtosis | *h^2^* |
| --- | --- | --- | --- | --- | --- | --- | --- | --- |
| E1 | 55.0 | 160.0 | 99.58±0.55 | 17.20 | 17.27 | 0.42 | 0.19 |  |
| E2 | 57.0 | 159.0 | 100.07±0.55 | 17.22 | 17.21 | 0.42 | 0.33 |  |
| E3 | 58.0 | 164.0 | 100.91±0.55 | 17.37 | 17.21 | 0.42 | 0.25 |  |
| CE | 55.0 | 164.0 | 100.18±0.32 | 17.27 | 17.23 | 0.42 | 0.26 | 0.95 |

*SE* Standard error; *E1 (Jiamusi_2017), E2 (Jiamusi_2018), E3 (jiamusi_2019) and CE (combined environment).*
